# Extreme energy expenditure during a 200 km three-day open-water swim: A doubly labeled water case study

**DOI:** 10.1016/j.jesf.2026.200528

**Published:** 2026-09-21

**Authors:** Bas Van Hooren, Gerard Rietjens, Guy Plasqui

**Affiliations:** a NUTRIM Institute of Nutrition and Translational Research in Metabolism, Maastricht University Medical Centre+, Department of Nutrition and Movement Sciences, Maastricht, the Netherlands; b Human Physiology and Sports Physiotherapy Research Group, Vrije Universiteit Brussel, Brussels, Belgium

**Keywords:** Energy expenditure, Physical activity level, Endurance exercise, Swimming, Doubly labeled water

## Abstract

The upper limits of total energy expenditure (TEE) in humans have primarily been described during single-day ultra-endurance events, or during multistage land-based endurance events across several days. However, little is known about the physiological limits of sustained energy turnover during shorter multi-day, near-continuous exercise events, particularly when performed in an aquatic environment. Prolonged immersion in water imposes substantial thermoregulatory demands due to increased conductive heat loss, which may further elevate metabolic requirements beyond those observed in (cold) land-based events. The 200 km Elfstedentocht swim, completed over approximately three days with minimal sleep and near-continuous swimming, provides a unique opportunity to investigate the upper limits of human energy expenditure during prolonged multi-day exercise in water. In this case study, we therefore assessed TEE in a former Olympic athlete (mass 103 kg, height 2.04 m, age 38 years) during a 200-km swim completed over 3 days using doubly labeled water. TEE was 195 Megajoule (MJ) (46,700 kcals), corresponding to 6.17x basal metabolic rate over the three-day period. This TEE is among the highest TEE reported thus far over similar periods, suggesting that the combined near-continuous exercise and imposed sustained thermoregulatory demands due to conductive heat loss lead to extreme energy expenditure. Notably, the absolute energy expenditure of ∼14,700 kcal per day exceeds previous limits.

## Key points

• The study documents energy expenditure during a 3-day aquatic endurance event where thermoregulatory demands differ from land.
• Total energy expenditure reached 195 MJ (≈47,000 kcal), corresponding to about 6.2× basal metabolic rate.
• At 14,700 kcal/day, this is the highest documented energy expenditure, suggesting human limits may be near or exceeded.

## 1 Introduction

Over short time periods, human energy expenditure can reach extraordinarily high levels. For example, single-day ultra-endurance competitions such as Ironman triathlons^1^ and 24-h mountain bike races^2^ have demonstrated that humans can transiently sustain extremely high rates of energy turnover with values up to 46 MJ (MJ) (∼11,000 kcal) per day 1, 2, 3. When this is expressed as a multiple of the basal metabolic rate (BMR; also referred to as a physical activity level [PAL] value) to account for differences in body size, reported PALs are up to fourteen times the BMR.^3^^,^^4^ Similarly, high PAL values of up to 5.3× BMR have been reported over longer endurance events such as the 23-day Vuelta a España^5^ and Tour de France.^6^

Exercise or physical activity in cold environments further amplifies metabolic demand because cold exposure increases thermoregulatory requirements and thus requires additional energy to maintain body temperature. Indeed, a PAL of 6.6 has been reported over a 10-day measurement period of the Antarctic Plateau to the South Pole traverse 7, 8, 9. Importantly, this sustained 6.6 PAL over 10 days exceeds levels reported during much shorter events in comparatively temperate conditions (3–34°C),^1^^,^^2^ illustrating that environmental stressors such as cold exposure can substantially augment total metabolic demand. Most studies on exercise in cold environments have however focused on exposure to cold air (for an overview see Table 2 in ^3^). Compared to air, water immersion presents a unique thermoregulatory challenge because of its higher thermal conductivity, resulting in a ∼3.3-fold greater heat loss for equivalent temperature.^10^ Indeed, even at moderate water temperatures, prolonged immersion can impose a continuous metabolic demand for heat production, thereby elevating energy expenditure beyond that required for exercise or physical activity alone. When combined with near-continuous exercise and minimal sleep, this may push sustained metabolic output toward the upper boundaries of human physiological capacity. However, energy expenditure during prolonged aquatic exercise remains undocumented.

The 200 km Elfstedentocht swim provides a rare opportunity to investigate energy expenditure during prolonged aquatic exercise. Completed over approximately three days with minimal sleep and near-continuous swimming, this event represents a distinct model of prolonged endurance stress in which locomotor cost and thermoregulatory demand are simultaneously elevated. In the present case study, we therefore assessed total energy expenditure during the 200 km Elfstedentocht swim using the doubly labeled water method. By quantifying total daily energy expenditure over the three-day period, we aimed to determine whether sustained multi-day open-water swimming could elicit energy turnover approaching or exceeding the highest values previously reported in humans for similar durations, and thereby provide new insight into the upper limits of sustained human metabolism under combined locomotor and thermoregulatory strain.

## 2 Case report

### 2.1 General study design

This study was designed as an observational case report quantifying energy expenditure during a 200 km multi-day open-water swim. The swim was undertaken in 2019 as a charitable initiative to raise funds for cancer research. Total energy expenditure was measured using the doubly labeled water (DLW) method across a 76-h period encompassing the complete event and energy intake was documented from food protocols. Food type, kilocalories per 100 g, and product size were noted by the support staff, and estimated from recipes for whole meals. Basal metabolic rate (BMR) was estimated using a prediction equation developed for trained athletic populations. All procedures were conducted in accordance with institutional ethical standards, and the athlete provided written informed consent.

One year prior (i.e., 2018) to the successful 200-km swim, the athlete made his first attempt at swimming the same distance, but had to give up after 55 hours and 163 km. During this initial swim, energy expenditure and intake were quantified using similar methodology as for the 200 km swim reported in the present case report.

### 2.2 Participant

The participant was a former Olympic-level swimmer (2008 Olympic champion 10-km open-water swimming) with extensive experience in ultra-distance swimming events. He for example also held the world record 24-h pool swimming until 2025. Notably, he also survived leukemia. At the time of the event, his body mass was 103 kg, height was 2.03 m, and age 38 years.

### 2.3 Event preparation

To prepare for the first (2018; unsuccessful) attempt, the swimmer swam approximately 10 hours per week over a two-year period. During this preparation, the duration of the longest training sessions was progressively increased from approximately 6 h to a maximum of two consecutive days of swimming.

Preparation for the second (2019; successful) attempt consisted of a four-month period of structured training, during which swimming volume was progressively increased. Key training sessions included a single 50-km swim, followed by two consecutive days of 50 km swimming per day (with sleep/rest in between), and culminating in four consecutive days of 50 km per day (approximately 14–15 h of swimming per day). From three days before the attempt, additional carbohydrate-rich snacks were consumed to maximize muscle and liver glycogen availability. No other specific nutritional preparation was implemented.

### 2.4 The exercise: 200 km Elfstedentocht swim

In both years, the athlete attempted to complete a 200 km open-water swim. The first, unsuccessful attempt took place in August 2018. During this attempt, the athlete aimed to complete the swim with minimal sleep, resting on only two occasions for approximately 20 min each over a total duration of 55 hours. Moreover, energy intake over the 55-h period amounted to only approximately 13,000 kcal. The combination of severe sleep restriction and a substantial energy deficit relative to the estimated energy expenditure (see later) likely contributed to the premature termination of the attempt after 55 h, at a total distance of 163 km.

One year later, the athlete successfully completed the 200 km open-water swim over approximately 73.5 hours with minimal (but more vs. the initial attempt) sleep and near-continuous forward movement. The athlete started swimming around 17:30 and swam until approximately 03:30. He then rested (attempted to sleep on a boat, in the drysuit, see below) for 1 hour before recommencing swimming until 01:00 the next day, where he slept until 04:00. The athlete then swam again until 01:00, slept until 04:00, and subsequently swam until 17:00 to finish the event (Fig. 1). Total rest time (including partial sleep) over the 73.5-h period was 7 hours.

**Fig. 1:**
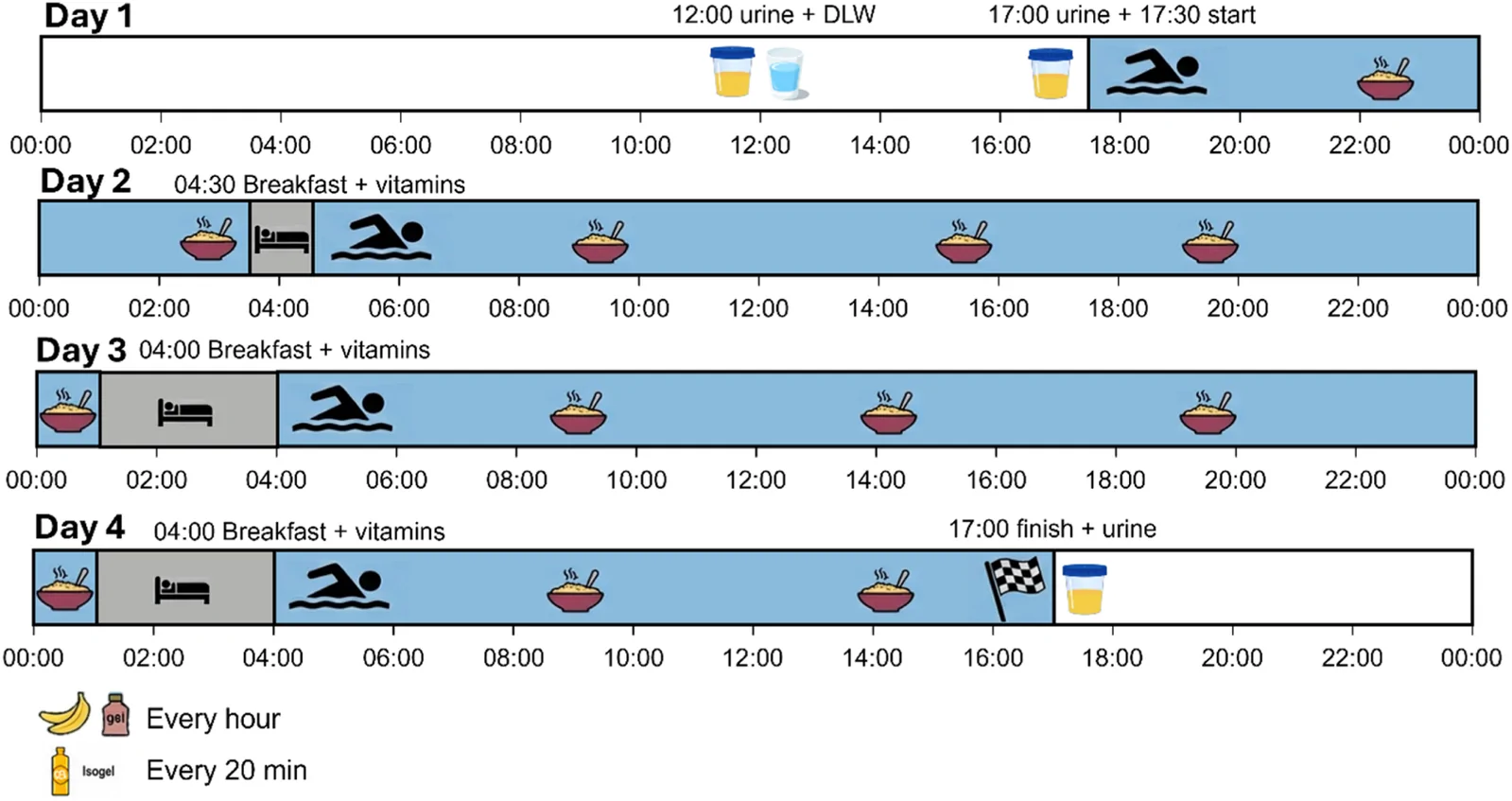
**Schematic of the swimming event.** Every hour, the participant consumed half a banana, a fiber gel, or muesli bar. Every 20 minutes, an isotonic gel was provided, and caffeine was also consumed after every hot meal. DLW = doubly labeled water.

The event was performed in open water of 20.5 degrees Celsius, thus imposing sustained thermoregulatory demands due to conductive heat loss. To minimize heat loss and ensure feasibility, the athlete wore a full-body wetsuit (including feet and hands) with an additional trunk-only dry-suit. Feeding occurred every 20 minutes during brief rest moments (<1 minute rest) where the support crew provided nutrition while the athlete remained in the water. Nutrition included carbohydrate gels, bananas, muesli bars, and 125 ml sports drinks. Larger meals were also provided every 4 h (e.g., potatoes with meat and red beets), with rest being 10-15 minutes for these larger meals. Total energy intake was approximately 10,000 kcal per day (∼30,000 kcal total). Stroke frequency was manually counted throughout the swim event and used to compute the total number of strokes made.

### 2.5 Energy expenditure and body composition assessment

Total energy expenditure was measured using the doubly labeled water method. To this purpose, the participant's body mass (fasted, in underwear) and height (no shoes, socks) were determined to prepare the dosage of doubly-labeled water according to “The Maastricht Protocol”.^11^ The water mixture contained 6.6 atomic percent ^2^H_2_O and 10 atomic percent H_2_^18^O, resulting in an initial excess body water enrichment of ∼150 ppm for deuterium and ∼300 ppm for oxygen-18. A baseline urine sample was taken 5.5 hours prior to the event, and the DLW dosage was then consumed by the participant from a small bottle of water (80-160 mL). To ensure that all isotopes were administered, after consumption the bottle was filled with 50-75 mL tap water, which was consumed as well. Urine was again collected 5 hours post dosing (from the second voiding), just prior to the event to calculate the isotope dilution space and the final sample was obtained within approximately 1 hour after finishing the event. Immediately after collection, urine was pipetted into 2 × 2 ml airtight glass vials and transported to Maastricht University where they were stored at −20°C until further analysis.

All urine samples were analyzed for the ^2^H and ^18^O concentrations as described by Plasqui et al.^12^ Briefly, the urine samples were equilibrated with H_2_ and CO_2_ gases, after which the enrichment of the two gases was measured by isotope ratio mass spectrometry.^13^ Dilution spaces of ^2^H and ^18^O were calculated using the plateau method, which uses the background enrichment in the baseline urine sample and the urine sample from the second voiding after equilibration.^14^ Total body water (TBW) was calculated as the average of both TBW-estimates (N_*d*_ and N_*O*_), with a correction for in vivo isotopic exchange. ${CO}_{2}$ production was calculated from the difference in elimination rates between deuterium (*k*D) and oxygen (*k*O) using the updated equation of Speakman et al.^15^ Energy expenditure was calculated using the Peronnet equation,^16^^,^^17^ while assuming a respiratory exchange ratio (RER) of 0.85, consistent with mixed substrate oxidation assumed previously for prolonged endurance exercise (for an overview see Supplemental table 2 in 18) and consistent with previously measured food quotients during prolonged exercise.^3^ An assumed RER was used instead of a measured food quotient because the nutrition intake details were not available anymore at the time of the data analysis for this study. Water turnover (rH_2_O) was estimated by using deuterium dilution space (N_d_) and its elimination rate (*k*_d_), applying the formula: rH_2_O = N_d_ x *k*_d_.^18^

Basal metabolic rate was estimated using the Ten Haaf equation,^19^ which predicts BMR in athletic populations based on fat-free mass. Fat-free mass was computed from TBW under the assumptions of fat tissue being free of water and fat-free mass having a constant water content of 73%.^20^ Because the Ten Haaf equation^19^ estimates basal metabolic rate based on fat-free mass, we considered this equation more accurate for the present study compared to equations developed specifically for elite endurance athletes that do not use body composition.^5^ Physical Activity Level (PAL) was calculated as total energy expenditure divided by basal metabolic rate.

### 2.6 Statistical analysis

Given the case-study design (n = 1), analyses were descriptive in nature, and no inferential statistics were performed. Outcomes are presented as absolute and body mass normalized values and daily averages where appropriate. Physical activity level and cumulative energy expenditure were derived directly from measured total energy expenditure and estimated BMR.

### 2.7 Results

Table 1 reports all subject details and primary outcomes for the successful attempt. Total body water (TBW) was 61.14 L. Estimated body fat percentage was 18.7%, giving a fat-free mass (FFM) of 83.8 kg. The calculated BMR was 10.0 MJ·day^−1^. Total energy expenditure during the 76-h measurement period between the pre-event sample to post-event sample averaged 61.7 MJ·day^−1^, corresponding to approximately 14,700 kcal·day^−1^. Cumulative energy expenditure over the full 76-h period was approximately 195 MJ (∼46,600 kcal). Using a BMR of 10.0 MJ·day^−1^ derived from the Ten Haaf equation, the calculated Physical Activity Level (PAL) was 6.17. This indicates sustained metabolic output exceeding six times the basal metabolic rate across three consecutive days.

**Table 1:** Subject details and primary outcomes for the successful 200 km swim.

Variable (Unit)	Value
Body mass (kg)	103
Body fat (%)	18.7
Fat-free mass (kg)	83.7
Total body water (L)	61.1
Measurement duration (h)	75.9
Average swimming speed, excluding rest periods (km·h^−1^)	3.01
N_d_/N_o_	1.034
Total daily carbon dioxide production (L·day^−1^)	2495
Total daily energy expenditure (MJ·day^−1^)	61.7
Total daily energy expenditure (kcal·day^−1^)	14,747
Cumulative energy expenditure over 76 h (MJ)	195
Cumulative energy expenditure over 76 h (kcal)	46,600
Total daily energy intake (kcal·day^−1^)	10,000
Cumulative energy intake over 76 h (kcal)	30,000
Estimated cumulative energy deficit (kcal)	16,600
Water turnover (L·day^−1^)	14,8
Basal metabolic rate (MJ·day^−1^)	10.0
Physical Activity Level (PAL, dimensionless)	6.17
Number of swimming strokes	200.000

After the event, the athlete rested/attempted to sleep and consumed a high protein diet for two days after the event to aid in the recovery of muscle damage. After the second attempt, the athlete did not experience a hunger kick, unlike the prior failed attempt to swim 200 km, suggesting better nutrition intake and energy balance in the successful attempt. The self-reported time for recovery of muscle soreness and overall recovery (including blisters) after the event were 3 and 14 days, respectively. Self-reported mental recovery (restoration of sleep pattern and sensitivity to external stimuli) took up to several weeks.

## 3 Discussion

This study provides the first directly measured estimate of total energy expenditure during a continuous ultra-endurance effort of approximately three days. Previous direct assessments of the upper limits of sustained human energy expenditure have relied either on doubly labeled water measurements obtained over substantially shorter (∼1 day *n* = 3^3^^,^^9^) or longer (≥5 day) durations, or on indirect estimates derived from energy balance and performance data. Moreover, none of the previous studies examined the upper limits of sustained energy expenditure during prolonged cold-water exposure while providing external nutritional support. Both factors may increase energy expenditure beyond that reported in studies conducted in temperate or cold air environments, and studies where participants were required to carry their own food supplies, potentially constraining energy intake. Consequently, the metabolic limits of human performance over intermediate durations have remained uncertain^9^. The present findings therefore fill an important gap in the literature by demonstrating that a PAL of ∼6.2 can be sustained during a 76-h (3-day) open-water swim. The observed magnitude of sustained energy turnover (PAL of ∼6.2) reflects extreme energy expenditure, but is lower than predicted by Thurber and colleagues,^9^ where a PAL of ∼9.5 would be predicted at 3 days. While this suggests even more extreme energy expenditure may be possible, this prediction is based on a combination of directly measured energy expenditure from doubly labeled water studies, but also estimates of energy expenditure based on energy balance and running speed during ultramarathon records, with the latter two approaches introducing potential for error. Our findings therefore provide the first indication of directly measured energy expenditure over such a duration, suggesting possibly lower limits than predicted from indirect estimates, yet still extreme magnitudes. Notably, the absolute energy use per day of 14,700 exceeds the highest value identified in a recent systematic review of 10,070 kcal,^21^ likely because of the large body mass (103 kg) of the present athlete.

The extreme energy expenditure in the present study likely reflects a combination of near-continuous exercise and additional energy requirements to maintain body temperature in the relatively cold moving water (∼20.5°). Indeed, moving water results in a ∼3.3-fold greater heat loss than moving air for equivalent temperature,^10^ which likely substantially increased energy expenditure above the exercise alone, despite the use of two wetsuits. However, the two wetsuits also increase resistance, thereby increasing energy expenditure for each arm stroke. The high energy expenditure in the present study was facilitated by the intake of substantial energy (∼10,000 kcal per day), with nutrition being provided every 20 minutes to minimize energy deficit. An important distinction from several previous studies in cold environments is that food reserves had to be carried by the individuals, which may have limited the food reserves and therefore energy intake 22, 23, 24. This contrasts with the present study, where food reserves were provided (Fig. 1), possibly facilitating higher energy intake and therefore expenditure.

One year prior to the successful completion of the 200-km swim, the athlete attempted the same distance but discontinued the effort after 55 h and 163 km. Retrospectively, several factors were identified that likely limited the success of this first attempt, including inadequate energy intake, gastrointestinal complaints, an overly aggressive swimming pace, and insufficient sleep (only 40 min over the entire 55-h period). These experiences informed modifications to the preparation and execution strategy for the subsequent 2019 attempt. Greater emphasis was placed on maintaining energy availability and incorporating opportunities for sleep, while adopting a pacing strategy that was more compatible with prolonged exercise duration. Consistent with these changes, energy intake during the successful attempt was almost doubled, increasing from approximately 5700 kcal·day^−1^ during the unsuccessful attempt to approximately 10,000 kcal·day^−1^. This greater energy availability coincided with a higher total energy expenditure during the successful attempt (∼14,700 kcal·day^−1^; PAL 6.2) compared with the initial attempt (∼12,900 kcal·day^−1^; PAL 5.5; quantified using the same methodology as in the present study) (Table 1). These observations suggest that sustaining extreme endurance performance requires not only a high capacity for energy expenditure but also a strategy that enables sufficient energy intake. Notably, because the nutrition provided was relatively low in fiber content, there were no gastrointestinal complaints during the second attempt. Furthermore, although sleep remained limited during the successful attempt, sleep duration was substantially increased compared with the first attempt. The successful attempt was also scheduled in June rather than August, providing longer daylight hours, which the athlete reported as beneficial for maintaining motivation and psychological resilience. Collectively, these adaptations highlight that successful completion of extreme multi-day endurance events depends not solely on maximizing effort, but on developing an integrated strategy that balances pacing, nutritional intake, sleep opportunities, environmental conditions, and psychological factors to allow continued performance over several days.

Body water turnover during the race (including the pre- and post-time) was 14.8 L per day, or 0.61 L·h^−1^ (Table 1). The hourly water turnover is lower than the 1.33 L·h^−1^ reported during other extreme endurance events in warmer conditions such as an Ironman triathlon.^25^ This difference is likely attributable to reduced thermoregulatory cooling (i.e., less sweating) demands during swimming in cooler water compared to the heightened thermoregulatory requirements of events in temperate or hot environments. However, despite the lower hourly turnover, the prolonged duration of continuous exertion resulted in a very high absolute daily water turnover, representing the one but highest value documented to date.^21^

In addition to extreme metabolic demands, the 200-km swim also induced extreme mechanical demands on the upper body. Indeed, the athlete almost exclusively relied on arm movement for propulsion, with approximately 200.000 strokes completed over the 200km swim. The highly repetitive arm movement along with one-sided breathing resulted in shoulder pain and blisters, which required the use of pain medication to facilitate completion of the event. The self-reported time for recovery of muscle soreness and overall recovery after the event were 3 and 14 days, respectively, further highlighting the high demands of the event. These observations are clinically relevant because they show that the health risks of such events are not limited to energy deficit or thermoregulation, but also include repetitive-load injury, pain management, skin integrity, and post-event recovery.

### 3.1 Limitations

Several limitations should be considered when interpreting the findings of this study. First, we had to make an assumption about the respiratory exchange ratio to compute energy expenditure from the calculated carbon dioxide production. The estimated energy expenditure is however relatively sensitive to assumptions of the respiratory exchange ratio, with an assumed respiratory exchange ratio of 0.8 increasing PAL to 6.48, and 0.9 decreasing PAL to 5.89. Nevertheless, all variations still yield extremely high energy expenditure values. The resulting PAL value is also sensitive —although to a lesser extent— to variations in the equation used to compute energy expenditure from the measured carbon dioxide production, with the PAL being 5.99 with the Weir equation. However, because the Weir equation was developed primarily for measurements of resting metabolism, we intentionally applied the Peronnet equation, which was specifically developed to account for altered substrate use during exercise. Second, we used the plateau method to estimate initial isotope dilution spaces, which in turn are used to calculate isotope turnover and energy expenditure. While this method is known to overestimate energy expenditure in high energy turnover,^14^ it was used for logistical reasons. Given the extreme duration and magnitude of the activity performed in this case, an overestimation would however unlikely alter the conclusion that the achieved energy expenditure was exceptionally high. Third, body mass was not measured after the event because prolonged water exposure resulted in substantial fluid retention and tissue swelling, potentially compromising the validity of body mass measurements. Finally, while nutritional intake was measured, insufficient details were available at the time of writing of this study to compute a food quotient for use in DLW data analysis.

## 4 Conclusion

The energy expenditure of >6x the basal metabolic rate is among the highest relative energy expenditures reported thus far over similar periods, with the absolute energy expenditure of ∼14,700 kcal/day being among the highest absolute energy expenditure every reported, suggesting that the combined near-continuous exercise and imposed sustained thermoregulatory demands due to conductive heat loss led to extreme energy expenditure. These findings also yield new insights from direct measurements into the upper range of human metabolic capacity over a 3-day period. More broadly, this case suggests that the practical upper limit of sustained human energy expenditure emerges from the interaction between metabolic capacity, environmental demands, and the effectiveness of nutritional and recovery strategies. For clinicians and exercise scientists, the case therefore offers a practical teaching example of how metabolic limits should be interpreted within the full context of event logistics and environmental strain, rather than as isolated energy-expenditure values alone. Future studies should therefore consider these factors together when investigating the limits of prolonged human performance.

## Data availability

Data is available upon request and is also available from the Doubly labeled water database.

## Competing interest statement

No external funding was received for this manuscript. The authors disclose no conflict of interest. All results are presented clearly, honestly, and without fabrication, falsification, or inappropriate data manipulation.
